# Enhancing Digital Health Awareness and mHealth Competencies in Medical Education: Proof-of-Concept Study and Summative Process Evaluation of a Quality Improvement Project

**DOI:** 10.2196/59454

**Published:** 2024-09-20

**Authors:** Fatma Sahan, Lisa Guthardt, Karin Panitz, Anna Siegel-Kianer, Isabel Eichhof, Björn D Schmitt, Jennifer Apolinario-Hagen

**Affiliations:** 1 Institute of Occupational, Social and Environmental Medicine, Centre for Health and Society Medical Faculty and University Hospital Düsseldorf Heinrich Heine University Düsseldorf Düsseldorf Germany; 2 Startup4MED, Dean's Office of the Medical Faculty Medical Faculty and University Hospital Düsseldorf Heinrich Heine University Düsseldorf Düsseldorf Germany

**Keywords:** medical students, digital health, design thinking, digital health literacy, medical education, digital health competencies, mobile phone

## Abstract

**Background:**

Currently, there is a need to optimize knowledge on digital transformation in mental health care, including digital therapeutics (eg, prescription apps), in medical education. However, in Germany, digital health has not yet been systematically integrated into medical curricula and is taught in a relatively small number of electives. Challenges for lecturers include the dynamic field as well as lacking guidance on how to efficiently apply innovative teaching formats for these new digital competencies. Quality improvement projects provide options to pilot-test novel educational offerings, as little is known about the acceptability of participatory approaches in conventional medical education.

**Objective:**

This quality improvement project addressed the gap in medical school electives on digital health literacy by introducing and evaluating an elective scoping study on the systematic development of different health app concepts designed by students to cultivate essential skills for future health care professionals (ie, mobile health [mHealth] competencies).

**Methods:**

This proof-of-concept study describes the development, optimization, implementation, and evaluation of a web-based elective on digital (mental) health competencies in medical education. Implemented as part of a quality improvement project, the elective aimed to guide medical students in developing app concepts applying a design thinking approach at a German medical school from January 2021 to January 2024. Topics included defining digital (mental) health, quality criteria for health apps, user perspective, persuasive design, and critical reflection on digitization in medical practice. The elective was offered 6 times within 36 months, with continuous evaluation and iterative optimization using both process and outcome measures, such as web-based questionnaires. We present examples of app concepts designed by students and summarize the quantitative and qualitative evaluation results.

**Results:**

In total, 60 students completed the elective and developed 25 health app concepts, most commonly targeting stress management and depression. In addition, disease management and prevention apps were designed for various somatic conditions such as diabetes and chronic pain. The results indicated high overall satisfaction across the 6 courses according to the evaluation questionnaire, with lower scores indicating higher satisfaction on a scale ranging from 1 to 6 (mean 1.70, SD 0.68). Students particularly valued the content, flexibility, support, and structure. While improvements in group work, submissions, and information transfer were suggested, the results underscore the usefulness of the web-based elective.

**Conclusions:**

This quality improvement project provides insights into relevant features for the successful user-centered and creative integration of mHealth competencies into medical education. Key factors for the satisfaction of students involved the participatory mindset, focus on competencies, discussions with app providers, and flexibility. Future efforts should define important learning objectives for digital health literacy and provide recommendations for integration rather than debating the need for digital health integration.

## Introduction

### Background

Initiated by the slow digital transformation of the German health care system, the German Federal Parliament passed the Digital Healthcare Act in December 2019, which made it possible to prescribe certain medical apps [1]. Since then, topics related to medical informatics, digital health, and telemedicine have started to appear more and more in the curriculum of some German medical schools, although mostly through few electives rather than compulsory subjects [2,3]. As digital health is a highly complex, dynamic field that constantly changes and advances and that has rarely been implemented into medical curricula [4,5], it seems necessary to compile and establish designated novel teaching formats that focus on the different subtopics, such as digital mental health interventions (DMHIs; eg, mobile health apps for dealing with depressive symptoms or managing study-related stress).

Digital mental health literacy, as well as competencies, becomes more and more important for (future) health professionals and medical education. It can be defined as “the degree to which individuals obtain, process, and understand basic mental health information and services needed to aid their recognition, management, or prevention of mental health issues” [6]. Even though younger generations are often supposed to be familiar with digitization and the corresponding competencies, research has shown that medical students do not feel adequately prepared for digitization in their course of study [2,3,7,8].

Previous studies have found that medical students perceive insufficient digital health literacy [9] and know little about available DMHIs [10,11]. At the same time, mental disorders have relatively high prevalence rates among the general population (ie, approximately 27.8% of the general German population have a mental disorder [12], and approximately 20% of the German population have depressive symptoms [13], a general decline in mental health in the last year [14]), and digital interventions could offer additional treatment and prevention options [15]. A systematic review and meta-analysis demonstrated that pooled depression prevalence among medical students worldwide was 37.9% [16]. DMHIs are especially relevant for medical students because, on the one hand, students are less likely to seek psychological help (due to barriers such as fear of stigmatization or lack of awareness) [17]. On the other hand, they themselves will eventually treat patients with (mental) health issues and may prescribe and use telemedicine, including digital health applications (in German: *Digitale Gesundheitsanwendungen* [DiGAs]) as future physicians [18].

Since October 2020, physicians and psychotherapists can prescribe DiGAs that are listed in the DiGA register by the German Federal Institute for Drugs and Medical Devices (Bundesinstitut für Arzneimittel und Medizinprodukte), including different DMHIs for mental disorders, on the expense of statutory health insurance companies [1]. DiGAs are certified medical products mainly based on digital technologies that can be used to detect, surveil, treat, or mitigate diseases, injuries, or disabilities [19]. The goals of DiGAs include the monitoring and improvement of current treatments in patient care. In the face of aging societies and the rise of chronic diseases, high-income industrial nations are confronted with rising health care costs. Therefore, digital health care and digital self-care practices are linked to efforts to better prevent disease, calculate disease risks and life expectancy through algorithm-based personalized medicine, and at the same time delegate clinical treatment responsibilities to the affected individuals themselves [20]. The potential of digital therapeutics or DiGAs in particular to improve the uptake of health care services has not been fully exploited yet, and the uptake is relatively low compared to prevalence rates [21]. In preventive medicine and disease monitoring, digital interventions could improve patients’ health and personal motivation [22]. In addition, digital data collection promises to optimize processes and increase the efficiency of the health care system at an institutional level [20].

Studies have shown that physicians are open to the idea of DiGAs [21], but the current prescription rates of DiGAs are low, with approximately 203,000 DiGAs prescribed or granted by health insurances in Germany [23]. Physicians in a mixed methods study described that they were skeptical (eg, due to technical insecurities) and said that they lacked adequate information sources on how to prescribe DiGAs and how to guide and advise patients concerning their use [19]. From a patient perspective, there is a clear interest in digital health as well. A recent German study on health app acceptance found that 76% of participants, including those without previous app experience, expressed willingness to use DiGAs [18]. Information measures can effectively increase acceptance of quality-assured digital health services among health care providers and patients [24]. To address the knowledge gap and enhance digital health competencies, practicing physicians are considering continuing education opportunities [21].

However, digital health literacy and digital competencies, or the acquisition of knowledge on DMHIs, including DiGAs for the treatment of mental disorders and the management of chronic conditions, need to be part of the curriculum, which could be piloted in elective subjects in medical schools. To the best of our knowledge, there are only a few studies concerning the teaching of digital health and digital competencies in German medical schools. Thus, little is known about strategies to implement such new teaching offerings in medical education. In one study, a total of 16 universities in Germany were identified that had included digital skills in their curricula (17 elective and 8 compulsory courses) [2]. For example, a study investigated the impact of an interdisciplinary and cross-faculty course concerning digital medicine with the help of a web-based questionnaire before and after the course [3]. Aulenkamp et al [2] found a positive impact of such courses on the students’ digital competencies and concluded that more efforts to integrate them into the curriculum would be necessary. One comparative study examined the implementation of a module on digital health among undergraduate medical students at a German university, the knowledge gain of students, and their attitudes toward digital health and suggested a firm implementation of digital competencies in medical education [25]. Another German study examined interdisciplinary teaching with the help of teaching teams of medical informatics professionals and physicians. In different academic years, new seminars on digital competencies were designed and implemented, and the usefulness of interdisciplinary teaching teams was demonstrated [26]. Further studies covered the integration of an elective on digital health in diabetes for pharmacy students [27] or the design, implementation, and evaluation of a course with a focus on telemedical components [28].

Overall, it is essential to permanently integrate digital health education into the curricula of medical schools [29,30]. Practical, competence-oriented didactic concepts offer promising strategies to enhance knowledge transfer and enable students to proficiently handle DMHIs and health apps.

### Goals of the Quality Improvement Project and the Case Study

The overarching objective of the quality improvement project was to develop and iteratively optimize an innovative learning and teaching offering for medical students based on their preferences and needs in a German medical school. The goals were targeted via an elective subject.

The elective subject in this proof-of-concept study aimed to provide students with basic knowledge and practical skills and promote a comprehensive understanding of designing concepts for digital health interventions in prevention and therapy with the potential user in mind. The focus of the elective was on digital health competencies in the field of mental health; the use of DMHIs in occupational and social medicine, especially in the area of primary prevention (eg, stress management); and DiGAs for somatic and mental diseases based on the students’ choices.

Our proof-of-concept study on quality improvement in medical education is meant to provide insights into the piloting of an innovative digital elective subject concerning the development of theoretical prototypes for DMHIs. We aimed to evaluate the implementation and realization of the elective subject using a design thinking process and analyze students’ feedback, ideas, and preferences concerning the competence-based education on mental health apps (“learning by doing” and cocreating).

## Methods

### Setting and Background of Teaching Innovation

The focus of the innovative teaching offering at a German medical school was on digital mental health in areas of application relevant to prevention and health care settings; quality criteria of mobile health apps; legal framework, including structural conditions for telemedicine (eg, the so-called Digital Healthcare Act in Germany and prescription of DiGAs); and user-oriented app design (eg, persuasive design [31]). The elective also aimed to promote knowledge of app development, self-competence, collaborative learning (codevelopment of an app concept in small student groups according to the prominent design thinking approach by the Hasso Plattner Institute [32]) as well as critical reflection on the opportunities and risks of digitalization for health professionals. The main target group were medical students, but due to the interdisciplinary nature of health app development, we allowed a small number of students from other disciplines to also participate.

### Quality Improvement Project

The quality improvement project was divided into 2 parts: funding (24 months) and the implementation of the web-based elective into the curriculum after the pilot project (12 months). The quality improvement project took place for 24 months from January 1, 2021, to December 31, 2022, at the Medical Faculty of Heinrich Heine University Düsseldorf (HHU) in Germany. We offered the digital elective 6 times within 3 years. The concept was based on a preceding elective subject involving a co-design workshop on digital mental health literacy in medical studies, which we conducted on campus with 26 medical students in March 2020, as described in sufficient detail by Dederichs et al [8]. In this subject, medical students developed theoretical prototypes of health apps in small groups using appropriate background knowledge and the established 5-step design thinking principle [33]. The students gave us feedback and indicated that they would have liked more flexibility, digital content in ILIAS (Integriertes Lern-, Informations- und Arbeitskooperations-System [German for “Integrated Learning, Information and Work Cooperation System”]), and additional guest lectures, which is why we developed the idea of a digital or hybrid implementation of the seminar (blended learning approach) [8]. The corresponding author conceived the project idea and acquired funding for an innovative educational project by the Commission for Quality Improvement in Teaching and Studies of the Dean’s Office of the Medical Faculty in November 2020. In January 2021, we prepared the digital elective “Fit for digitalization and ‘apps on prescription’?—Understanding digital health applications and developing digital health offers such as health apps” for the summer semester of 2021. Medical students were able to take the elective subject with 2 semester hours per week. Over time, we also extended the elective to students from other fields if there were enough available openings. This was achieved either in the framework of “studium universale” or as a psychology minor, including credit points for active participation. Finally, the following 12 months (January 2023-January 2024) were used to implement the project into medical education practice without additional staff, which required some adaptations, including providing a screen cast (ie, video tutorial) on the technical creation of app mock-ups using the collaborative design tool Figma (Figma, Inc) instead of personal assistance for each group.

### Structure and Composition of the Elective

We varied the elective either as an intensive block course (5 consecutive days, full time during the lecture time, and provided once) or as weekly seminars (7 times, part time during the lecture-free time, and provided 5 times), equating to a time effort of 2 semester hours for both options. For more intensive support, we increased the proportion of synchronous live lectures after the first run in 2021. In addition, students were able to access the content (eg, presentations) and additional material via the web-based learning management system ILIAS (ILIAS open source e-Learning e.V). Students could request feedback on their group work via ILIAS. We refined the ILIAS environment steadily to ensure its immediacy and to meet students’ preferences.

**Figure 1:**
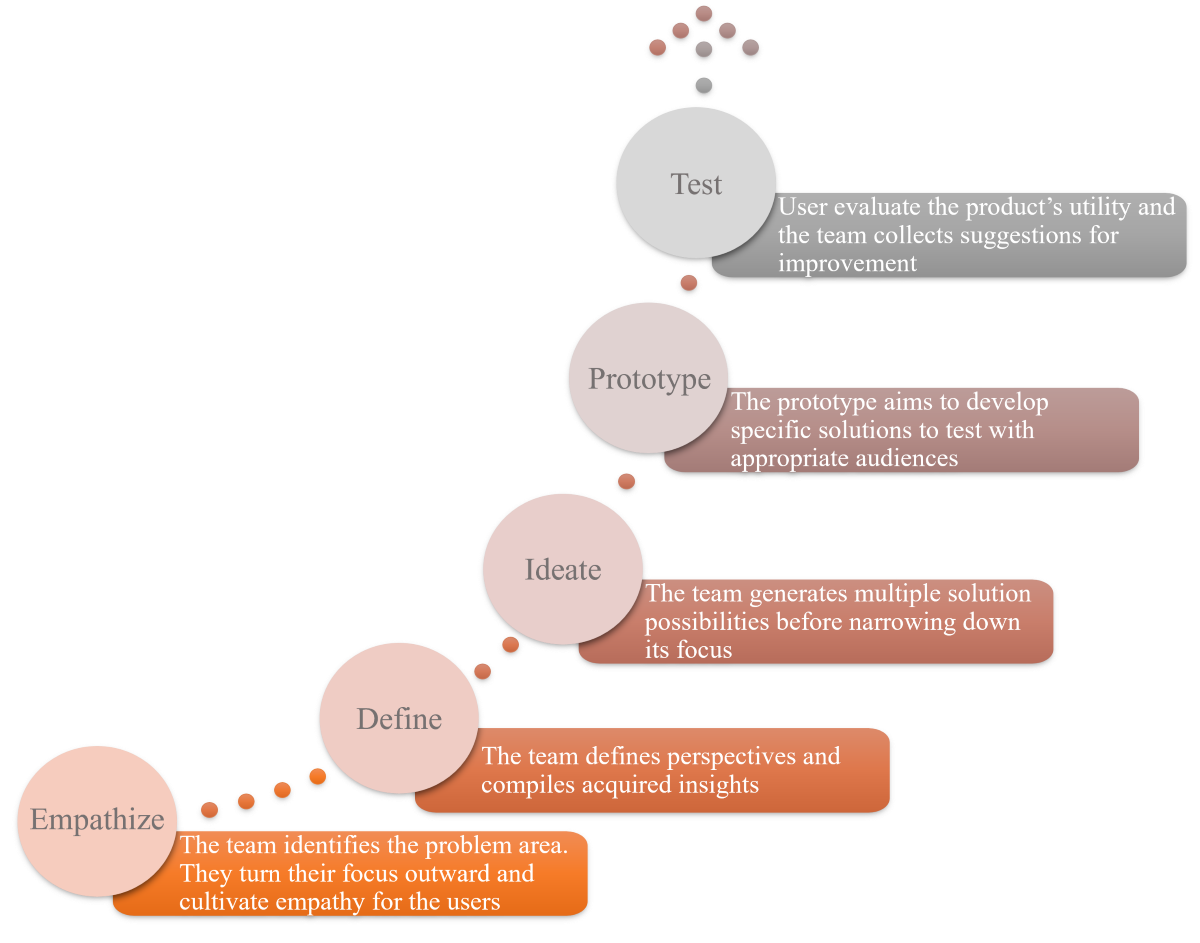
A simplified explanation of the 5 stages of design thinking (based on the work by Meinel et al [31]).

The focus was on competence-oriented learning and participatory design approaches. The acquired knowledge about health apps was directly transferred into a concrete app concept in the group work. In creating the group assignments, we were guided by the concept of design thinking and its phases for developing an app. Design thinking is an iterative, user-centered approach to innovation and problem-solving. This framework emphasizes the comprehensive analysis and understanding of human needs to generate original approaches to solving complex problems [31-33]. The process, which typically involves multiple stages, includes understanding the problem, carefully eliciting user-specific requirements, generating ideas, creating prototypes, and testing solutions (Figure 1 [31]).

The elective included an introductory session at the beginning of the semester, which introduced the procedure, contents, and objectives and brought students together in small groups with different app topics (eg, insomnia). Initial content-related issues were also covered (eg, definitions and fields of application). In lessons 2 to 5, the students were given a group task aligned with the content of the lessons and a design thinking process, which systematically guided them in the development of a hypothetical app.

A detailed description of the structure of the elective can be found in Table 1.

**Table 1:** Lessons and main contents of the elective course^a^.

Session number	Main topic	Contents
1	Organizational matters and introduction	Overview and examination Presentation of group results (app concepts) from previous semesters Introduction—working definition of digital mental health Areas of application Support with digital health interventions Evidence of e–mental health Telemedicine and digital health in occupational medicine Division into small groups for the development of own app concepts and introducing tools for group work First introduction to design thinking Option of test access to DiGAs^b^ (GAIA AG)
2	Quality criteria of health apps	Description of quality seals for health apps Description of quality principles Presentation of the DiGA register Quality measurement using the MARS^c^ [34] (German version [35]) Presentation of the search for quality-assessed health apps and e–mental health interventions Presentation of an app (eg, blood pressure app Manoa) Group work
3	DiGAs—“apps on prescription”	Regulation of the apps listed in the DiGA directory Acceptance and use of DiGAs Various guest lectures on mental health DiGAs (eg, Somnio, Deprexis, Elona Therapy Depression, and Velibra DiGAs) Guest lecture ReHappy (former stroke DiGA), including screencast (ie, video files of recorded lectures) Group work
4	Design thinking and persuasive design (app development)	Description of the phases of the design thinking process Presentation of concept mapping Teaching persuasive design and persuasive design in mental health apps Discussion on the “ideate” phase in the design thinking cycle and idea generation Incentives for managing successful apps Group work
5	Strategies to promote acceptance and adherence	Measures to promote user adherence and motivation by communicating acceptance models, such as the UTAUT^d^ and UTAUT2^e^ adapted to digital health [36,37] Presentation of usability in health apps (eg, via the SUS^f^ [38]) Description of gamification approaches for health apps Presentation on promoting acceptance through information Description of the distinction between health and medical apps as well as trusted health apps Group work
6	Finish for the final presentation	Explaining peer feedback Guest lecture from Startup4MED, HHU^g^ (each semester) Tips for the final presentations and peer feedback Group work
7	Final presentation of the group work	Lecture—summary plus current developments and perspectives Presentation of the developed app concepts in small groups Feedback and evaluation of the elective

^a^Detailed description of the elective structure (sessions 1 to 7). The contents partly varied across the semesters based on expert availability (digital health app providers) and adaptations made following the evaluation results of the former semester.

^b^DiGA: digital health application.

^c^MARS: Mobile App Rating Scale.

^d^UTAUT: Unified Theory of Acceptance and Use of Technology.

^e^UTAUT2: extension of the UTAUT to the consumer context.

^f^SUS: System Usability Scale.

^g^HHU: Heinrich Heine University Düsseldorf (Germany).

### Practical Transfer Through the Integration of Guest Lectures and Test Access

We provided students with selected guest lectures by experts who created, provided, or presented DiGAs to the professional community (eg, Somnio, Velibra, and Elona Therapy Depression). In addition, at the end of the course, we hosted lectures from staff members of the medical-specific start-up support unit “Startup4MED.” Startup4MED is the internal start-up support unit of the University Medicine Düsseldorf and identifies, promotes, and supports the commercial exploitation of innovative medical projects from the Medical Faculty of HHU and the University Hospital of Düsseldorf. This allowed participants to connect with the start-up support team for extra guidance on their ideas and on how to implement their app concepts in practice.

Each semester, students had the opportunity to attend between 1 and 3 guest lectures, some of which were recorded as screencasts (ie, videos in terms of recorded lectures) and uploaded online. In addition, students were given the chance to try free demonstration versions of various DiGAs through a trial program sponsored by a German company (GAIA AG) in 2021 and 2022.

### Student Support and Competence-Oriented Performance Recording

During their elective, students received comprehensive assistance through multiple media and communication channels that aligned with their individual preferences. These channels comprised Rocket.Chat, an open-source team chat platform, as well as Microsoft Teams. To reinforce their learning process, the participants were assigned concise, structured tasks following every session, and the completion of these tasks was monitored by the project team. We also offered personalized feedback and guidance on demand beyond the group feedback each week upon completion of the tasks uploaded using ILIAS. As part of the overall design thinking process, the students were gradually and systematically introduced to the app concept. This was done by providing weekly synchronous web-based lectures in addition to the educational material provided via ILIAS. This approach aimed to promote collaboration, reduce inhibitions to reach out, and provide students with more flexibility. In the last session of the elective, student groups presented their developed app concepts to their instructors and peers and received feedback. Optionally, the assessment could be completed as an exam. Finally, the students were instructed to evaluate the elective through a web-based survey provided via “evasys” (evasys GmbH).

### Evaluation

In addition to the oral feedback in the last session, we used a web-based evaluation questionnaire created using templates provided by the Dean’s Office (Department of Evaluation, Medical Faculty). The questionnaire, implemented using “evasys,” included standardized questions on digital teaching offerings and was completed by students online after finishing the course. Participation was voluntary, and thus, we tried to increase it by sending reminders. In the first part of the questionnaire, we asked students to provide information about their gender and their field of study. In the second part of the questionnaire, we asked students to rate the module through 19 questions. The first 13 were answerable on a 6-point Likert scale ranging from 1 (“completely agree”) to 6 (“do not agree at all”). These questions concerned the content of the elective as well as the visualization and access to ILIAS, such as “The learning module was well structured.” Finally, we assessed the perceived difficulty level of the learning material, overall satisfaction with the elective (both in general and regarding digital implementation), and the students’ own estimated learning gains after completing the elective.

A total of 3 additional questions dealt with the difficulty of the learning content, the scope of the learning and reading material, and the assessment of particularly helpful elements in the course (5 response options, eg, text units and screencasts). The last 3 questions served as feedback and were presented in the form of open questions (eg, what students liked most about the course, which competence area they benefited most from, and which suggestions for improvement they had). The evaluation was centralized and anonymized by the Dean’s Office. Results of the evaluation were provided in an aggregated format by the Dean’s Office if a minimum of 5 completed surveys per elective were available. Following the completion of the elective in the summer semester of 2021, the questionnaire was revised and used in the subsequent courses. We further analyzed quantitative data from the elective descriptively (eg, means and proportions) and summarized qualitative data (comment fields) using Microsoft Excel and SPSS (version 27.0; IBM Corp). Answers to the open-ended questions and comments were analyzed using MAXQDA 2020 (VERBI GmbH). We then formed categories both deductively based on our questionnaire and inductively from the material. The preliminary code system was discussed, revised, and agreed upon.

### Ethical Considerations

This proof-of-concept study received an ethics approval for retrospective analyses by the IRB of the Medical Faculty at the Heinrich Heine University Düsseldorf (ref. no. 2024-3033). We obtained informed consent for the publication of mockup figures designed by the student groups. The evaluation in this quality improvement project was conducted in a regular teaching context. The participation in the web-based survey on the subjective evaluation of the elective was voluntarily and conducted anonymously by the central evaluation of the study dean office upon completion of the elective, so that the authors had no access to personal data linking individual survey responses to specific participants. We also had no access to information on individual data such as gender, age, study subject or semester based on the aggregated results of the web-based survey. The web-based survey did not address sensitive topics and the respondents were not considered as a vulnerable group according to the nature of the survey as we only ask for them to indicate their views on the quality of the attended elective and optionally provide suggestions for improvements.

## Results

### Sample Characteristics

A total of 75 students (women: n=45, 60%) from HHU registered for the elective in 6 seminars over 3 years (2021-2024; ie, 5 semesters [no elective in summer 2023 due to parental leave of the project lead]). Of these students, 72% (54/75) were medical students (median 5, range 3-11 semesters), 12% (9/75) were psychology students (bachelor’s program; median 7, range 5-21 semesters), and 4% (3/75) were economics students (bachelor’s program; median 9, range 7-16 semesters). In addition, 1% (1/75) studied business administration (bachelor’s program; semester 1); 5% (4/75) were bachelor’s biology students (mean 9.50, SD 2.96 semesters; range 5-13); 1% (1/75) studied art history (semester 17), philosophy (semester 9), or medical physics (semester 7; bachelor’s program each case) each; and 1% (1/75) were master’s biology students (semester 1). Overall, 20% (15/75) of the students dropped out of the elective, mainly before its start, as they did not attend the first session. The remaining 80% (60/75) of the students successfully finished the elective (including course achievement). Of these 60 students (women: n=36, 60%), 77% (46/60) were medical students (median 5, range 3-11 semesters), 15% (9/60) were psychology students (bachelor’s program; as mentioned above), and 8% (5/60) studied one of the aforementioned subjects (2/5, 40% studied economics; median 12.5, range 9-16 semesters; and 1/5, 20% studied a bachelor’s biology program; semester 13; art history, and a master’s biology program).

### Insights Into Common Themes and App Development From the Sessions and Group Discussions

According to individual preferences, students focused on both mental health promotion and dealing with mental and somatic diseases in the development of the hypothetical app, but for this case study, we only reported some examples of health apps. After the first round of the elective, the scope was expanded beyond DMHIs in the course description for upcoming electives to better meet the preferences of more medical students. Overall, 25 projects were finished and presented, 10 (40%) of which covered somatic conditions and 15 (60%) of which covered mental health conditions or indications. The students were asked to create a name for their app concept and to check whether this app name exists already. The task was to create a suitable, recognizable name they can explain with respect to the hypothetical product and target group. However, we did not control each app name in terms of brands or specific products worldwide as this was an elective for educational non-commercial purposes. Table 2 shows an overview of all app concepts developed by the students in the different semesters.

The following main topics emerged in the prototypes of the apps: exam anxiety in students, stress management, resilience, insomnia and sleep disturbances in children and adolescents, and depression in youths and young adults as themes for mental health apps, with some frequently chosen topics (especially stress management). Furthermore, various disease management or prevention apps for somatic conditions were conceptualized (breast cancer, diabetes, stroke and arterial hypertonia, reflux disease, musculoskeletal diseases, glioblastoma, skin diseases, and premenstrual syndrome). Recurring app topics among different electives were stress, exam anxiety, and stress-inducing learning behavior regarding procrastination, as well as depression and anxiety.

In the context of app development, the students systematically applied the knowledge they had learned in the elective subject considering various aspects, guided each week on different aspects by the elective’s team (5 steps of design thinking [31,33]). The topics that students emphasized most often in their presentations of their app concepts included target group-specific information (eg, prevalence rates and relevance of the app for health care); usability; and features of persuasive design, including gamification, accessibility, and the promotion of adherence (eg, through reward systems, gamification, provision of human support, and cost reimbursement), which we will illustrate with suitable case examples in the following sections. The featured functionalities varied depending on the selected app concept. For apps related to disease management, students often incorporated symptom diaries, reminders about medications or physician’s appointments, and educational resources about the corresponding illness. In contrast, apps aimed at stress management during learning focused mainly on structuring daily routines, relaxation, and avoiding procrastinating behaviors. An example of this would be the ability to lock smartphones for a set period. Most app prototypes included considerations for integrating professional guidance (eg, via chat functions). These chat functions either established contact with professionals or facilitated communication among user groups.

**Table 2:** Overview of the different app concepts in each semester partially translated into English.

Number	Topic or indication of the app	Target group or population	Name of the app concept	Semester
1	Chronic conditions, diabetes	Patients (adults)	My Diabetes Pass	SS^a^ 2021
2	Learning support, self-management, stress management	Pupils and students	Studytime	SS 2021
3	Resilience promotion	Young people in education	Mental Power	SS 2021
4	Stress reduction	Students	MeTime	WS^b^ 2021-2022
5	Musculoskeletal diseases, pain	Patients (primarily adults)	legLos (“getStarted”)	WS 2021-2022
6	Sleep disturbances, insomnia	Patients (children aged 3-12 years, supported by their parents)	Morpheus goes to sleep	WS 2021-2022
7	Depression, depressive symptoms	Patients (aged 13-25 years)	Dinotherapy	WS 2021-2022
8	Gastroesophageal reflux disease	Patients (aged >40 years)	StopGERD	WS 2021-2022
9	Breast cancer	Patients (adult female individuals)	BRUHNO	WS 2021-2022 intensive block
10	Stress reduction, exam anxiety	Students and trainees (aged 15-30 years)	Companion	WS 2021-2022 intensive block
11	Glioblastoma	Patients (aged 50-70 years)	GlioblAPP	WS 2021-2022 intensive block
12	Stress reduction, stress prevention	Company employees (ie, finance sector)	Stress Cutter	WS 2021-2022 intensive block
13	Back problems, pain	Patients (aged 18-60 years)	Backfit	SS 2022
14	Eating disorders	Patients (ie, anorexia nervosa)	Provida	SS 2022
15	Hypertension, blood pressure problems	Patients (mainly adults aged >50 years)	Eutonia	SS 2022
16	Stress management after rehabilitation	Patients (adults)	iGrow	WS 2022-2023
17	Blood pressure problems, arterial hypertension	Patients and risk groups (adults)	Tonus	WS 2022-2023
18	Sleep disturbance in depression, burnout	Patients (adults aged 35-45 years)	Happy Sleeper	WS 2022-2023
19	Exam anxiety	Students (aged 18-25 years)	Exam Anxiety	WS 2022-2023
20	Skin diseases	Patients (children and adults)	DermaDiary	WS 2023-2024
21	PMS^c^, PMDD^d^, self-management	Patients (mainly female individuals aged 18-30 years)	ZenCycle	WS 2023-2024
22	Self-management, (emotional) self-regulation	Primary school children (their parents or legal guardians)	MaxiKids	WS 2023-2024
23	Stress management	University students	UniRelax	WS 2023-2024
24	Resilience promotion	Health care staff, nurses (aged 16-64 years)	Pflege-Care (ie, care for nurses)	WS 2023-2024
25	Depression, depressive symptoms	Patients (adults)	Livetta	WS 2023-2024

^a^SS: summer semester.

^b^WS: winter semester.

^c^PMS: premenstrual syndrome.

^d^PMDD: premenstrual dysphoric disorder.

### Case Examples: App Development and Concepts

In this section, several app prototypes that were developed by student groups are presented. All prototypes chosen for this paper serve as comprehensive and visually clear examples of app concepts. All design samples, images, and the creative theoretical work belong to the students and cannot be used without their permission.

**Figure 2:**
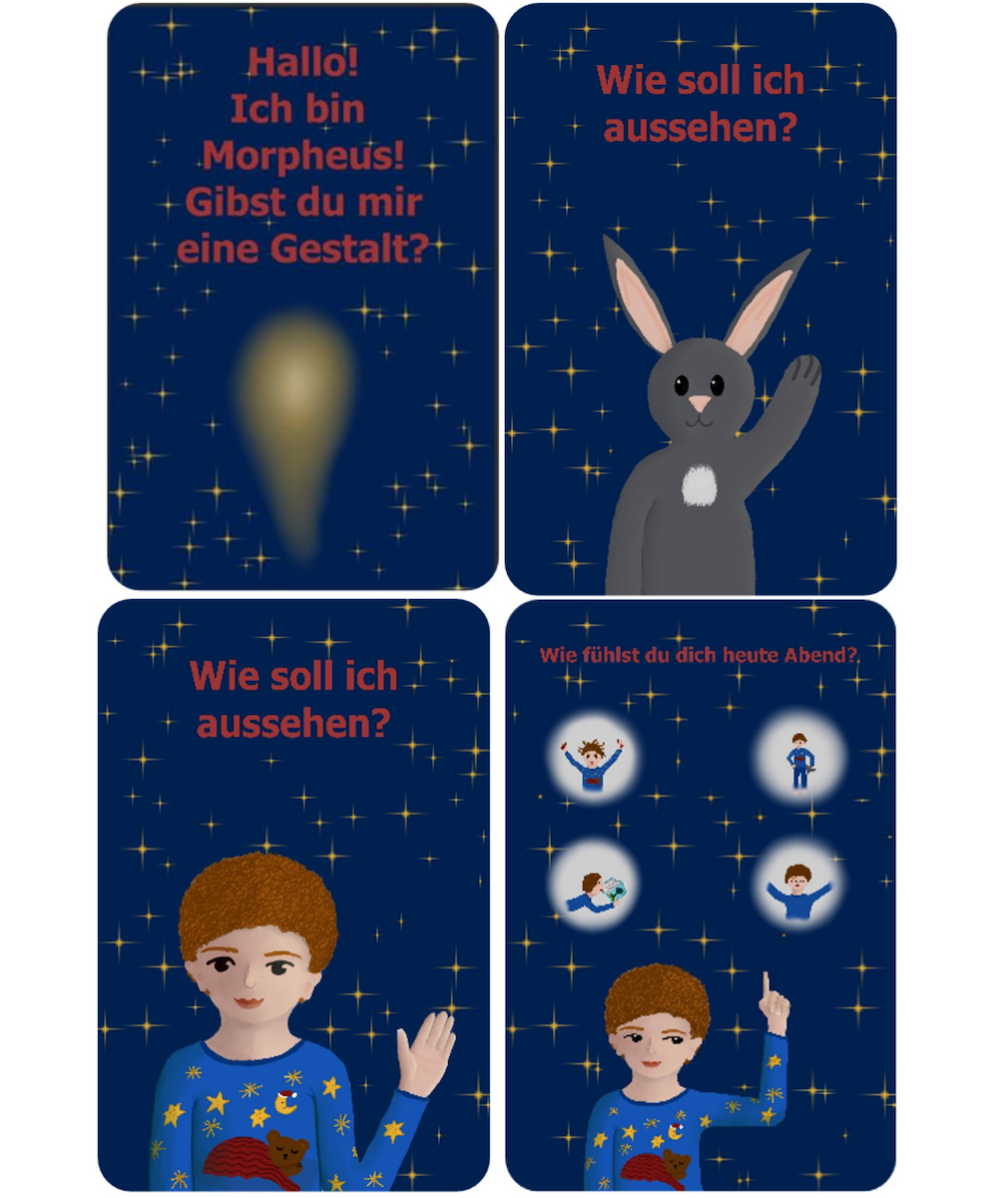
Mock-ups of the Morpheus geht schlafen (Morpheus goes to sleep) app. Top left: welcome screen. Top right and bottom left: choice of the customizable companion (animal or human). Bottom right: assessment of individual well-being.

Morpheus geht schlafen (Morpheus goes to sleep) is an app for sleep disturbance in children. A group of 2 students (n=2, 100% female) created this app concept to improve sleep hygiene and facilitate falling asleep for children. In Textbox 1, the app and its design and implementation are described in detail. Figure 2 visualizes the app concept.

**App concept for Morpheus geht schlafen.**

**Morpheus geht schlafen (Morpheus goes to sleep) app concept**

- Students developed the app for (nonorganic) sleep disturbances in children.
- The selected target group was children aged between 3 and 12 years.
- Concerning design and aesthetics, students chose dark background colors, little movement on screen, a background that remains the same, and a dark color scheme with little white. They also added a customizable companion (human or animal) with rounder body shapes to make it more appealing for children and to present a positive body scheme.
- According to the students, the app can be used to improve sleep hygiene and facilitate falling asleep and could be adapted in relation to the child’s level of activity. Methods included exercises concerning movement and relaxation, fantasy journeys with progressive muscle relaxation, bedtime stories with integrated breathing instructions, “sound forests,” and lullabies.
- Additional functions covered a link to the app alarm (default setting for when to get up, individualization by parents, and calculation of sleep phases and the ideal sleep time), a sleep tracker, and, optionally, a companion toy with speaker function.
- Students said that parents should be involved via either a separate app or a button for parents. Parents could be supported and informed about sleep hygiene in children (examples concerning a child-oriented evening routine, information on sleep disturbances in children, and a section with frequently asked questions).
- Concerning adherence promotion, students thought of the individualization of the companion, fun facts when brushing teeth, interesting activities, and the collection of stars for every use day. Nonadherence should also be detected (notifications in case of nonactivity).
- Data security was another very important aspect for the students. They explained that the app should include a data privacy statement. In addition, the use of the app is only possible after an active confirmation of informed consent. Data are stored on the device, and data transfer needs are separately authorized (or data can be downloaded as a PDF file).

**Figure 3:**
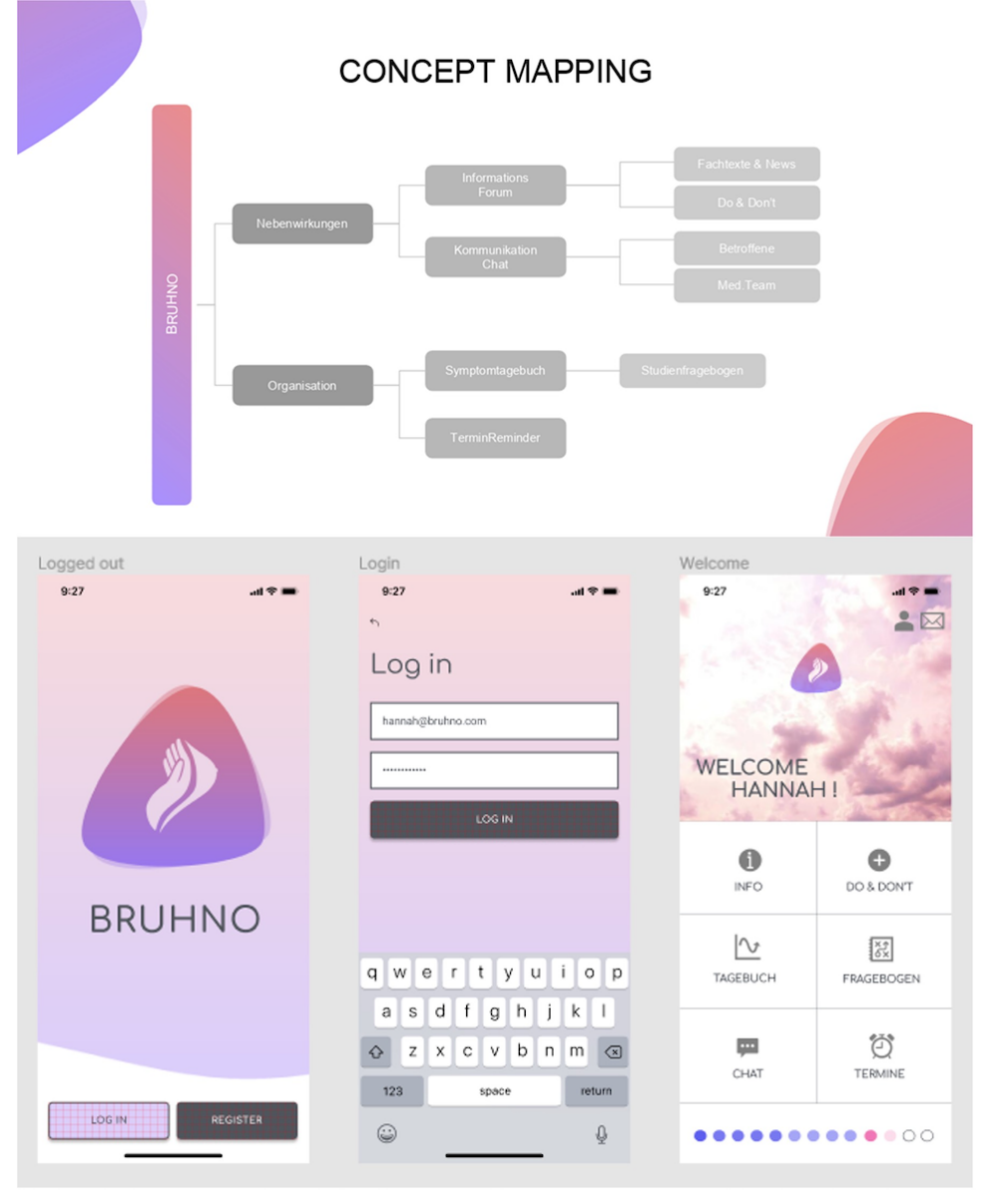
Mock-ups of the Bruhno app. Above: concept mapping for the Bruhno app mock-up as developed by the students. Below: mock-up of the Bruhno app-logo and log-out, log-in, and welcome screens.

Bruhno—Brustkrebs Helfer für Nebenwirkungen und Organisation (Breast cancer helper for side effects and organization) is a companion app for female patients with breast cancer. A group of 2 students (n=2, 100% female) created this app concept to support women in systemic therapy and aftercare. In Textbox 2, the app and its design and implementation are described in detail. Figure 3 visualizes the app concept (for additional prototypes, see Figures S1 and S2 in Multimedia Appendix 1).

**App concept for Bruhno.**

**Bruhno app concept**

- Students developed the app as a companion for female patients with breast cancer (support in systemic therapy and aftercare).
- The selected target group was predefined (participants of a clinical study with a diagnosis of breast cancer). Female patients aged ≥18 years (mean age 40-65 years) with sufficient technical abilities and equipment (mobile phone and internet access) should use the app.
- The following goals and functions were intended by the student group: reduction of fear (chat with physicians or other persons affected and short explanatory texts or videos), enhancement of well-being (eg, tips to manage side effects), data collection and monitoring (query of side effects, symptom diary, possible evaluation by physician, and questionnaires on life quality), appointment reminders (push notifications, and appointments could be entered by the clinic team or the patient), and an optional intake tracker in case of oral medication.
- Included methods were information, education, data collection, monitoring, tracking, and reminders.
- In the concept of the app, factors to promote adherence were tracking progress using a timeline and maintaining personal motivation through social exchange with other app users via chat.
- The student group also considered technical aspects related to the use of the app. Internet access is vital, and additional web applications would be useful to type longer posts or contributions. In addition, aspects concerning certification, advertisement, and financing were considered (categorization as a medical product and sale to study organizers or sponsors).

**Figure 4:**
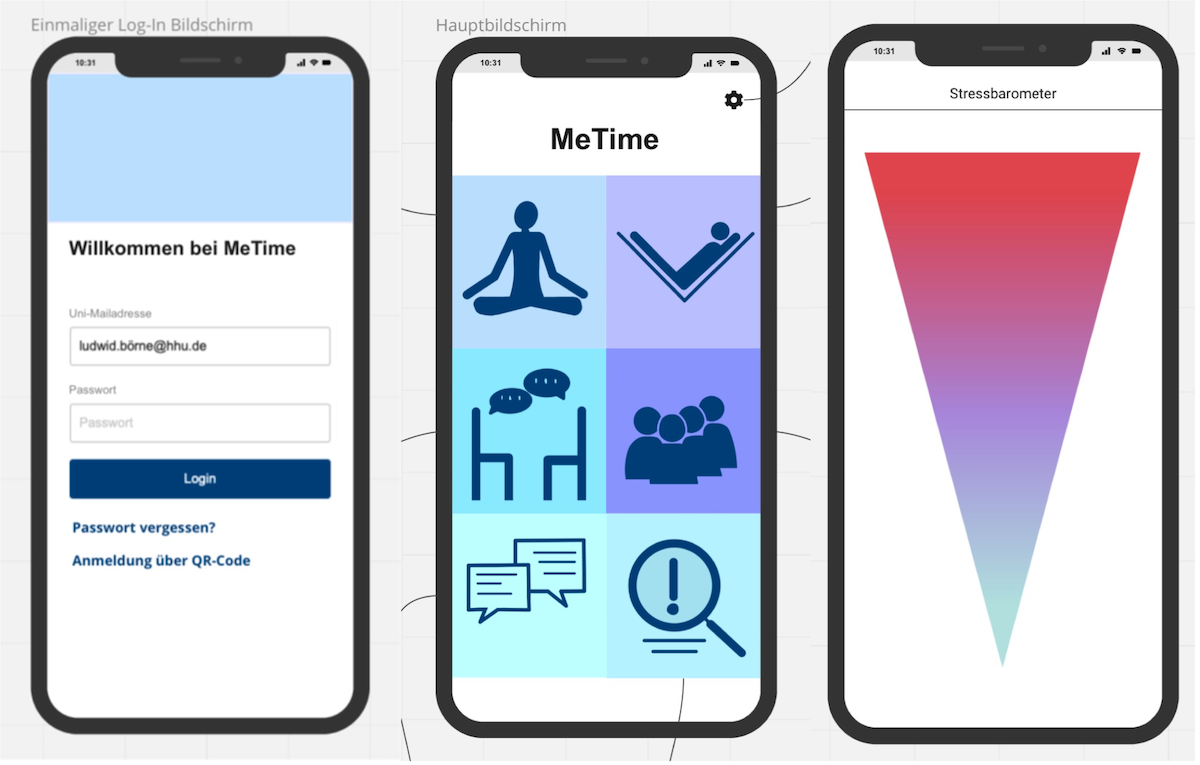
Mock-ups of the MeTime app. Left: one-time log-in screen. Middle: welcome screen. Right: visualization of a stress barometer.

MeTime is an app for stress reduction in students. A group of 3 students (n=3, 67% female) created this app concept to promote relaxation in students. In Textbox 3, the app and its design and implementation are described in detail. Figure 4 visualizes the app concept (for additional prototypes, see Figures S3 and S4 in Multimedia Appendix 1).

**App concept for MeTime.**

**MeTime app concept**

- The app MeTime was developed by a group of students to reduce stress and promote relaxation for students.
- The relevant target group were students from Heinrich Heine University Düsseldorf in acute phases of stress (eg, exam periods).
- The concept of the app was to provide relaxation exercises and possibilities for networking and social exchange.
- Factors related to the design were also considered by the students. The app should be intuitive and minimalistic. Appealing colors, little text and many symbols, and a stress barometer should also be implemented.
- Students also added a companion, Heinrich, to the app (referring to the first name of a German writer and poet and eponym of the Heinrich Heine University Düsseldorf). This companion supports users, serves as a search engine for the lexicon, reads out instructions and texts, and wears different clothes depending on the context or situation.
- Use options and options to personalize the app were considered. Stress reduction, networking, and education were seen as important. In addition, personal exercises can be created and selected, favorite exercises can be chosen, and an anonymous chat can be used. Color scheme and text size should be customizable, and the user’s personal calendar can be accessed. Individual stress levels should be assessed by the user before and after an exercise.
- Concerning app costs, students suggested including the costs in the semester fee. The chosen category for the mHealth app in app stores was lifestyle, health, and fitness.

**Figure 5:**
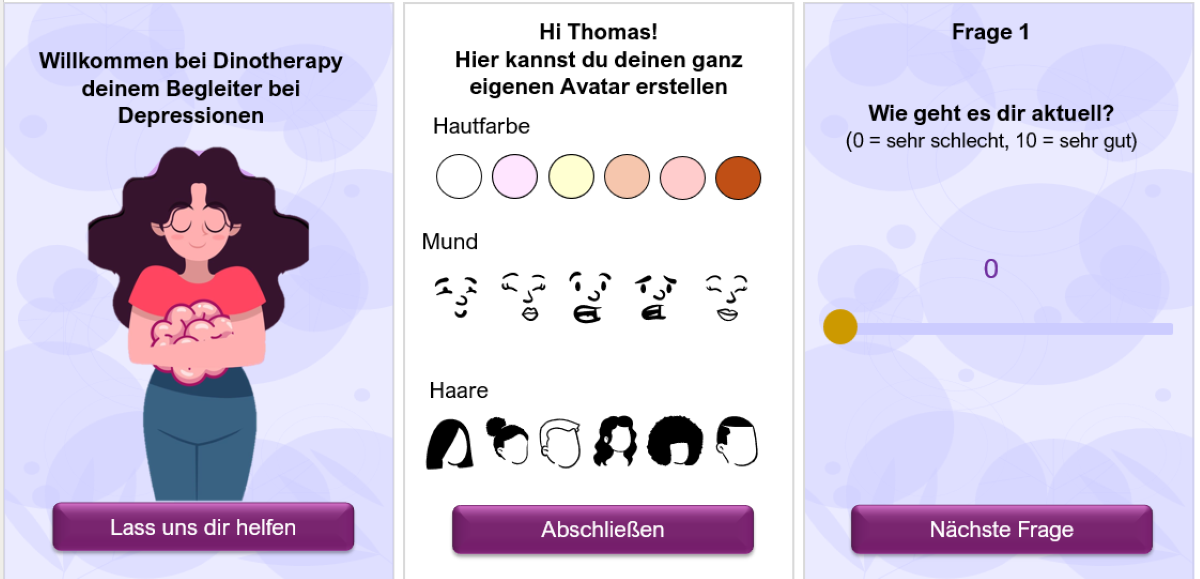
Mock-ups of the Dinotherapy app. Left: welcome screen (vivid user interface to create a positive user experience). Middle: options for the individual, customizable avatar or companion. Right: questionnaire to assess the user’s mood.

Dinotherapy is a companion app for depressive episodes in youths and adolescents. A group of 2 students (n=2, 100% male) created this app concept to bridge the time until psychotherapy starts or as a companion during psychotherapy. In Textbox 4, the app and its design and implementation are described in detail. Figure 5 visualizes the app concept (for additional prototypes, see Figure S5 in Multimedia Appendix 1).

**App concept for Dinotherapy.**

**Dinotherapy app concept**

- Students developed the app as a companion for youths and adolescents who are diagnosed with depression.
- The selected target group were youths aged between 13 and 25 years.
- The app combines interactive provision of psychoeducational content (using gamification elements) with practical tasks and uses dinosaurs as protagonists, identification figures, and “virtual pets.”
- According to the students, the app has different goals: (1) development of knowledge and understanding of the disease—“depression as a disease,” (2) continuous tracking of the course of the depression, (3) establishing activities and fixed rituals that will have a beneficial effect on the course of the depression, and (4) social exchange with other persons affected via the app.
- Students said that a link between the accounts of the patient and physician or psychotherapist responsible can be possible. This way, the attending person could set priorities; assign patients to each other as peers; and specifically be informed if depression values fall below a critical threshold in case, for example, there is a suicide risk.
- To promote adherence, students thought of self-monitoring (tracking using short, daily questions), a visual presentation of the course of depression, tailoring and personalization of tasks and contents, rehearsals or reminders, appraisal and points for each completed task that can be used for the dinosaur (the “virtual pet”), and peer interaction.
- The design of the dinosaurs is also meant to promote adherence and should be appealing for the selected target group.
- The following functions were included by the students: (1) surveys (*TRACK*), (2) comics (*EDUCATE*), (3) tasks (*ACT*), (4) interaction (*EXCHANGE*, and (5) *INTERVENT*.

**Figure 6:**
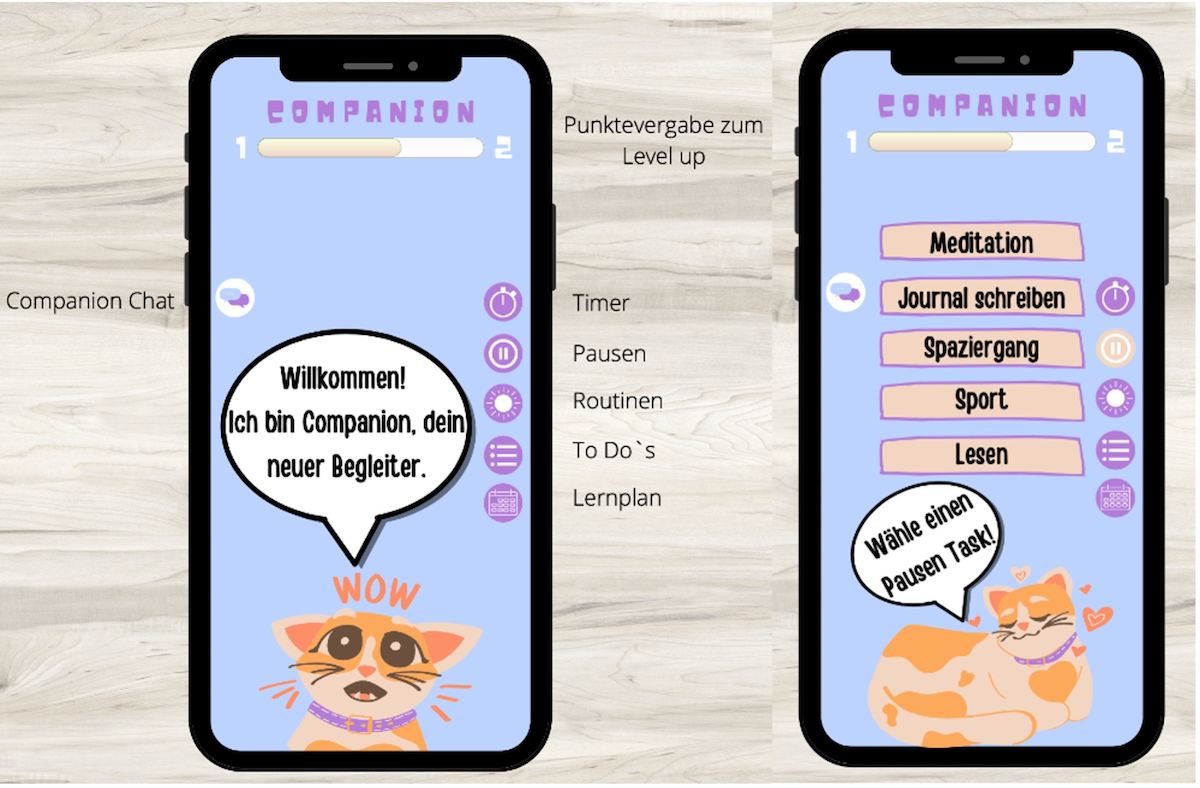
Mock-ups of the Companion app. Left: welcome screen, current level, functions, and the customizable pet. Right: selection of tasks and activities for breaks.

Companion is an app to reduce stress and exam anxiety and enhance support for students and trainees. A group of 2 students (n=2, 100% female) created this app concept to accompany the target group in the course of the study and in vocational training. In Textbox 5, the app and its design and implementation are described in detail. Figure 6 visualizes the app concept (for additional prototypes, see Figures S6 and S7 in Multimedia Appendix 1).

**App concept for Companion.**

**Companion app concept**

- Students developed the app as a companion for students and trainees. The selected target group were users aged between 15 and 30 years.
- Students introduced an individual pet that accompanies users and explains the handling of the app. They also implemented daily individual and evidence-based hints to reduce stress, exam anxiety, or procrastination.
- In the app, learning plans, to-do lists, and morning and evening routines can be created. In addition, motivating reminders are used. Chatting with the companion (artificial intelligence) is possible. Further functions are structuring, a timer, time management, and the blocking of other apps to work productively.
- To promote adherence, the students implemented progress confirmation, the collection of points for leveling up, and rewards for new levels (eg, unlocking new items for the companion pet) in the app concept. They also paid attention to personalization and adaptation regarding user needs (tailoring).
- To enhance credibility, the students suggested the following: universities should recommend the app, physicians and therapists should test and also recommend it, scientific researchers should be involved in the app development, and the app should receive a seal of approval.
- No data are meant to be given to third parties. If an account is deleted, all individual data are also deleted, and there is anonymous feedback.
- Unique selling points developed by the students were rewards for breaks and not only for work phases (to promote a healthy balance between resting and working), the combination of various functions in 1 app, and evidence-based psychoeducation for users’ daily lives.
- The app is meant to be a free app in the app stores or an app with a monthly subscription and a free test version (eg, for 14 days). University licenses and licenses from training facilities should enable use free of charge.

The theoretical prototypes developed as part of the elective course were designed with a strong practical orientation and regard to applicable national requirements for medical devices. This opens up the possibility of using the products designed in the course in the inpatient or outpatient medical sector in the future.

The Bruhno and Dinotherapy student groups approached Startup4MED with their ideas concerning the app concepts. Together with the start-up support unit, the students were given the opportunity to plan and work on the transfer of their theoretical prototypes into practice. For this purpose, next steps, analyses of marketability and customer needs, and financial requirements were examined in individual consultation sessions. To address the financial requirements for further development of the concepts and their implementation into initial prototypes, suitable funding was identified, and the application process was supported. Providing contact with founders of successful medical start-ups, additional team members, and mentors from the Startup4MED network further aimed to achieve translation of theory into practice.

The app concepts developed during the course showed valuable potential for the students to found their own start-ups and generate future-oriented innovations in the field of mental health. In addition, combining education and training in the field of digital health and the practical application of the designed products can make an important contribution to sustainable benefits in the field of innovation.

### Evaluation and Suggestions for Improvement

#### Quantitative Results: Questionnaires

Students were asked to complete a questionnaire that had been designed by the research team to assess the elective and receive suggestions for further improvement. To ensure comparability between the individual semesters, a basic average for each semester from all questionnaires submitted per elective was calculated (a total of 6 seminars within 5 semesters). Overall, 73% (44/60) of the students completed the evaluation survey.

**Figure 7:**
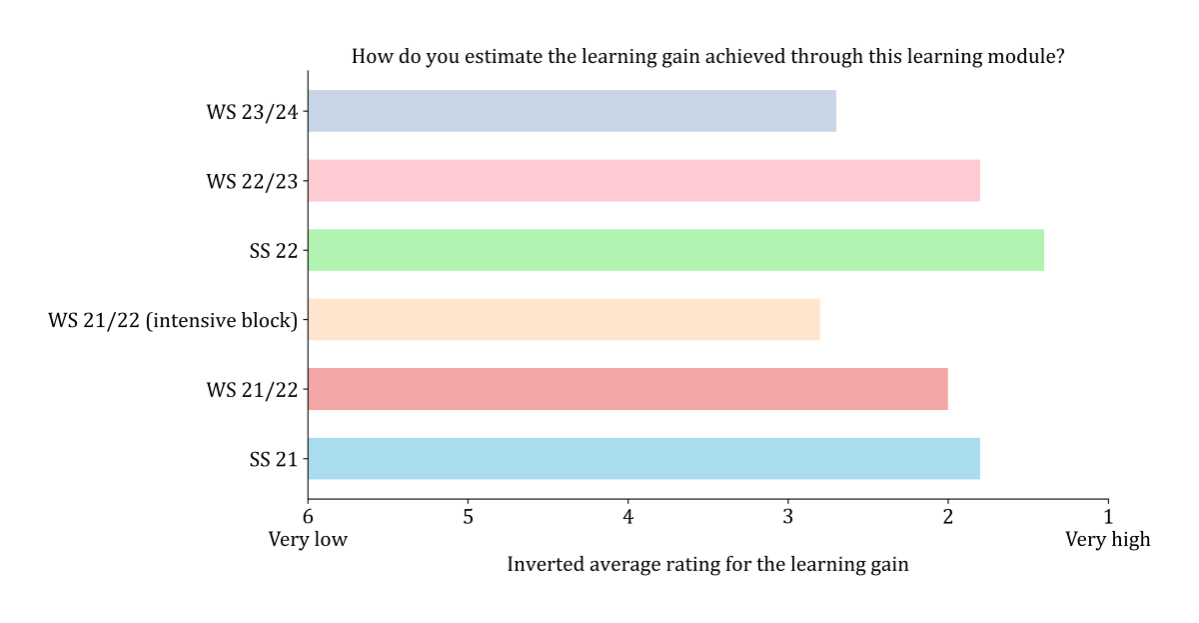
Assessment of students’ perceived learning gain from the elective conducted at the end of each semester. SS: summer semester; WS: winter semester.

**Figure 8:**
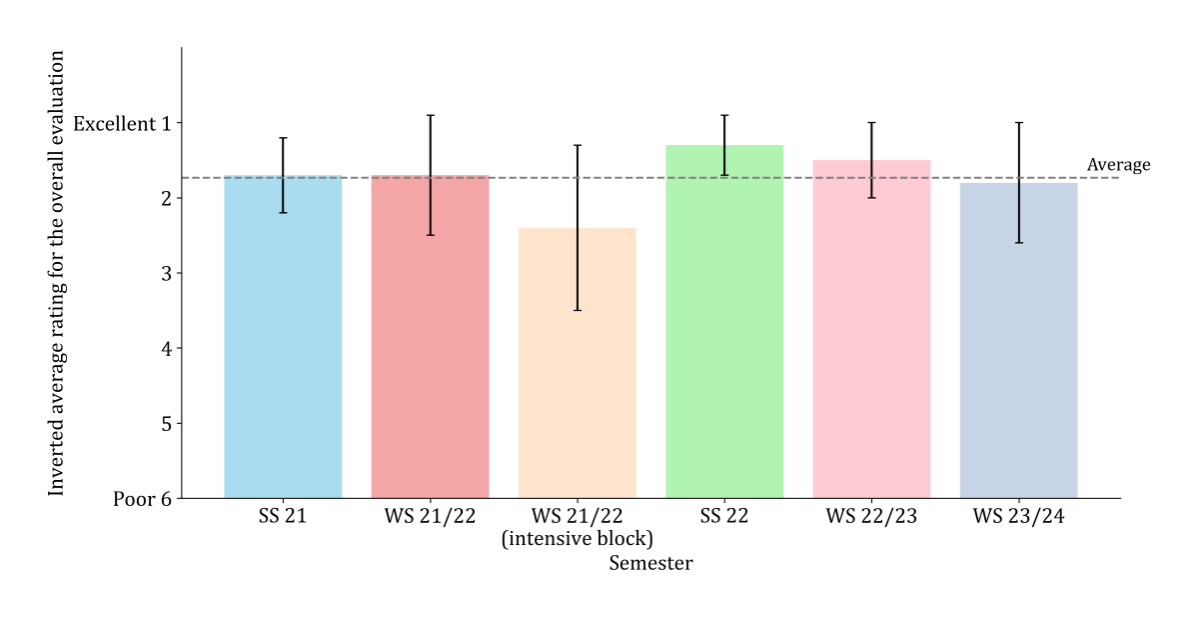
Overall evaluation of the elective subjects over 6 courses within 5 semesters. The ratings are based on student ratings in accordance with the German grading system (1=completely agree; 6=do not agree at all), with lower values indicating better ratings. The inversion is intended to improve readability, with higher bars now corresponding to higher satisfaction ratings. The dashed line indicates the average rating across all semesters. SS: summer semester; WS: winter semester.

Table 3 shows the results of the different items of the questionnaires for each semester. Figures 7 and 8 display the perceived learning gain and the evaluation of the elective overall (for additional results on the satisfaction with the module as well as the evaluation of the digitalization, see Figures S8 and S9 in Multimedia Appendix 2).

**Table 3:** Items from the evaluation questionnaire, including means and SDs per course^a^.

Item from the evaluation questionnaire	Course					
	SS^b^ 2021 (n=5^c^), mean (SD)	WS^d^ 2021-2022 (n=11), mean (SD)	WS 2021-2022 block seminar (n=9), mean (SD)	SS 2022 (n=5), mean (SD)	WS 2022-2023 (n=5), mean (SD)	WS 2023-2024 (n=9), mean (SD)
Overall assessment of the elective	1.50 (0.50)	1.70 (0.80)	2.40 (1.10)	1.30 (0.40)	1.50 (0.50)	1.80 (0.80)
Digital implementation	1.70 (0.80)	1.70 (1.10)	1.80 (0.70)	1.20 (0.30)	1.40 (0.60)	1.60 (0.70)
The learning module was well structured.	1.60 (0.50)	1.50 (0.70)	1.60 (0.70)	1.00 (0.00)	1.00 (0.00)	1.90 (0.90)
The content was made easy to understand.	1.20 (0.40)	1.60 (0.90)	1.80 (0.80)	1.00 (0.00)	1.40 (0.50)	1.30 (0.50)
The distinction between crucial information and insignificant particulars became evident.	2.00 (1.00)	2.10 (1.00)	3.40 (1.60)	1.50 (0.90)	2.00 (0.70)	2.10 (1.10)
The significance of the material instructed for the medical field became evident.	1.00 (1.00)	1.50 (1.00)	2.70 (1.10)	1.40 (0.50)	1.40 (0.50)	1.70 (0.70)
The visuals (such as interactive images and video tutorials) made it easier to grasp the educational material.	1.60 (0.90)	1.40 (0.50)	1.80 (0.80)	1.60 (0.50)	1.20 (0.40)	1.80 (0.80)
The integrated tests (eg, quizzes) were aligned with the learning content.	2.40 (0.80)	—^e^	—	—	—	—
The integrated tests (eg, quizzes) helped me check my understanding of the learning content.	2.40 (1.30)	—	—	—	—	—
Concrete tips were provided for following up on the learning material.	1.20 (0.40)	1.50 (1.20)	1.60 (0.70)	1.20 (0.40)	1.50 (0.50)	1.60 (0.70)
I found it easy to stay motivated throughout the module.	2.20 (1.30)	2.10 (1.50)	2.90 (1.20)	1.00 (0.00)	1.60 (0.90)	1.80 (1.20)
Access to the learning module was successful without any issues.	1.20 (0.40)	1.80 (1.50)	1.30 (0.50)	1.00 (0.00)	1.00 (1.00)	1.20 (0.40)
The learning module was easy to use.	1.20 (0.40)	1.50 (0.70)	1.30 (0.50)	1.00 (0.00)	1.40 (0.90)	1.40 (0.50)
All participants were engaged in the course.	—	1.70 (0.80)	1.80 (1.00)	1.60 (1.30)	1.20 (0.40)	1.80 (1.30)
There were various opportunities available for inquiries and exchanges (eg, through Rocket.Chat, Webex, ILIAS, or email).	—	1.20 (0.60)	1.40 (0.50)	1.40 (0.90)	1.00 (1.00)	1.20 (0.40)

^a^With regard to the first 2 items listed in the table, students were requested to provide an assessment of the elective subject in terms of the German school grades. A 6-point Likert scale ranging from 1 (“completely agree”) to 6 (“do not agree at all”) was used for the subsequent items.

^b^SS: summer semester.

^c^Questionnaires submitted per course.

^d^WS: winter semester.

^e^These items were subsequently removed from the questionnaire, consequently they were only surveyed for SS 2021.

The general feedback in the different semesters showed that the elective was well received by the students. The relevance of the various contents for their future work life was noticeable for many students, and there were enough opportunities to ask questions and discuss issues. It was also positively mentioned that the contents were explained in a clear and comprehensible way, that the learning module could be accessed easily, and that the elective was well structured. Room for improvement became apparent (eg, with regard to self-motivation, the perceived helpfulness of the integrated tests and quizzes, and the differentiation between important contents and less important detailed knowledge).

In the winter semester of 2021 to 2022, when the elective was presented as an intensive block seminar on 5 consecutive days, the overall rating and the rating concerning digital implementation were the lowest (Table 3) compared to those in the other semesters. However, the mean score was good in terms of German school grades even in the worst-rated intensive course. Concerning the intensive block seminar, students were less satisfied with the block course but still rated it as good. They reported having difficulties motivating themselves to do the tasks and complete the module.

#### Qualitative Results: Open Questions

In the open questions, students could give additional feedback (eg, suggestions for improving the elective) in the form of free text. Overall, many of them seemed to appreciate contents concerning digital health and challenges in implementing digital health offerings and perceived a substantial knowledge gain after taking part in the elective. Students liked the autonomy, flexibility, support from teaching staff, contents, and structure best:

Autonomy and free time management. Interesting content that is otherwise not covered in your studies! I learned a lot. Immediate feedback from the teaching staff, absolutely great!

Summer semester 2021

The entire elective offered us the opportunity to freely shape our ideas, but it was all within a professional framework.

Winter semester 2021-2022

Great support!

Summer semester 2022

Freedom of choice for the app concept.

Winter semester 2023-2024

Additional factors that were perceived as positive were creativity, the fact that they could always ask questions, the guest lectures, fixed submission dates, and social exchange:

The freedom to design and be creative yourself.

Winter semester 2021-2022

Plenty of room to ask questions.

Winter semester 2021-2022 intensive block

Permanent availability of the teaching staff.

Summer semester 2021

The guest lectures were very interesting.

Winter semester 2023-2024

When being asked which competence area students benefited most from, several participants mentioned knowledge gain, self-sufficiency, and soft skills. It was also important to assess perceived deficits of the elective and aspects that could be improved. Students mentioned that submissions and arrangements within the work group seemed to be improvable (eg, submission deadlines could have been communicated more clearly right at the beginning, and communication between group members should be enhanced):

Addressing clearer deadlines at the beginning: For me, it was a bit difficult to understand the structure with the respective modules etc. right away.

Summer semester 2021

Some participants perceived group work as exhausting because of the structure of the groups (ie, group members were determined based on thematic interests and it did not always seem to be clear enough who else was part of the group) and different work attitudes. It was also mentioned that some more face-to-face sessions would have been helpful (eg, to address problems and issues and to get in contact with the other group members). More information on how to realize and implement app ideas and concepts was also desired:

I would have liked more guest lectures to learn more about their experiences in app development.

Winter semester 2022-2023

How do you actually turn a concept into an app? Who else do you need for this; how do you get the app into the app store when it’s nearly finished?

Winter semester 2021-2022

Some students from the block seminar in February 2022 (winter semester 2021-2022) would have liked to have more time for the contents dealt with as it seemed to be a lot of input in a short period for them:

It’s a lot of input for one week. Maybe you can try to skip or cut some parts if possible.

Winter semester 2022-2023

Furthermore, it was said that the elective would have been preferred as an in-class or face-to-face event to facilitate exchange and discussions:

For me, it would be easier to follow the content of the course in person. Unfortunately, I drifted off a lot of times.

Winter semester 2022-2023

Concerning missing contents or topics, the following suggestions were made: more economic contents (eg, financial aspects, approaching sponsors, and approaching programmers), more information on how to turn a concept into an actual app or how to publish the app in an app store, and more guest lectures.

In the winter semester of 2023 to 2024, the issue of artificial intelligence (AI) was also brought up:

The topic, or presentation on AI in the medical setting was something new, I would have liked more of that. In general, more variety would be good.

Winter semester 2023-2024

## Discussion

This study describes findings in the piloting and iterative optimization of a new, digitally mediated elective subject in the field of digital health at a German medical school. A central objective was to involve students actively and continuously in the optimization and design of the elective from the beginning onward and successfully convey relevant content.

### Principal Findings

Through multiple iterations, a participatory, optimized, innovative teaching offering was developed tailored specifically to the needs and preferences of medical students. The overall implementation, digital execution, knowledge increase, content relevance, and supervision received (very) positive ratings by the students. The overarching goal of acquiring competencies and knowledge in the field of digital health (digital skills) was clearly achieved according to the surveyed participants.

Students considered several important aspects related to digital health (especially health apps, including DiGAs) and implemented them accordingly in their app prototypes. As previous studies have shown, gamification [39], adherence promotion, and an appealing design are important elements for creating a positive user experience [40,41]. These features were also considered by many student groups in our elective subject. However, it is important to emphasize that one should not only rely on elements of gamification to develop a useful, appealing, and persuasive app [41]. This was also considered by most of the students in their app concepts as they often included background information, useful functions depending on the purpose of the app, and information on data security. As data security concerns are one of the main barriers to the acceptance and broad implementation of mobile health apps, addressing these issues in the process of the app’s development is essential [42].

According to the participants, the expansion of their knowledge in the area of mobile health apps was successfully realized as they reported having more information and an improved understanding of digital health after completing the seminar. In particular, the opportunities for independent and creative work seemed to convince the students of our approach to teaching digital health and digital competencies. Examining the mean scores for digital delivery revealed a slight positive trend. The comprehensive ratings of 4 courses ranged from “very good” to “good plus” in terms of overall implementation and digital delivery. Interestingly, the block course (on 5 consecutive days) in the winter semester of 2021 to 2022 received substantially lower ratings than the other courses during the semester despite higher investment in the supervision. Potentially, the intensive course involved a workload perceived as too high with too little time between the sessions to develop the app concepts in a creative process that may benefit from a longer time to discuss the ideas within the group. Feedback regarding improvement suggestions indicated that the content and development of an app concept required more time for processing, which negatively affected student satisfaction with the elective in the form of a block seminar.

We were able to implement the proposed improvements, which proved successful as these suggestions were not mentioned again in subsequent course evaluations. However, it must be noted that there might be other possible reasons for this (eg, students with other needs in the following courses or students with generally lower expectations or previous knowledge). Some students emphasized in their feedback that the topics covered filled a gap in their previous education. This perceived gap aligns with the results of elective subjects in the field of digital health at other universities. Before participating in the digital health elective, a survey was conducted at another German medical school, the Charité–Berlin University Medicine, where >85% of participating students stated that digital medicine was not sufficiently integrated into the current curricula [25]. A further study examining medical students’ perspectives indicated their desire for a more robust integration of digital health into the curriculum [43]. While certain German medical faculties have initiated the provision of digital health electives [2], the integration of digital health topics into medical education in Germany and Europe is still not firmly established [9,26,44]. During our last elective course, the topic of AI was suggested for the first time. We had already anticipated this and included it as an excursus, which was well received by the students during the seminar. Current research indicates that most physicians and medical students have a favorable attitude toward the integration of AI into medical education. Many are already studying AI or intend to do so. The introduction of AI into the curriculum must be carefully planned to ensure that students’ education remains up-to-date. The digitalization of the health care system, the use of digital health apps, and AI in medicine are interconnected. Therefore, medical curricula should be adapted to the digital age as soon as possible [45].

However, contrary to expectations, no substantial increase in the number of participants, reflecting the demand for digital health education, was observed. In all semesters, the total number of participants in the elective course remained below the maximum capacity (5 to 17 students who successfully completed the elective). One exception was the winter semester of 2021 to 2022, in which the course was offered both weekly and as a block course during the lecture-free period with 30 course places, which was the upper limit of participants. This could be attributed to the wide range of parallel electives offered at the Medical Faculty at HHU (approximately 150 different electives each semester [46]) as well as the lack of integration of digital health into the National Competence-Based Learning Objectives for Undergraduate Medical Education. Higher registration numbers for other electives indicate that students’ interest and focus rather seem to lie on clinical diseases or imaging procedures, possibly due to a supposedly clearer and more tangible practical relevance. Feedback of the students in the final discussions after their app concept presentations also revealed that the content of the elective was considered very important but that there were other decisive factors in course selection, such as the integrability into the rest of their schedule and the expected workload. The number of participants should be further improved in possible subsequent courses after the intended integration of digital competencies into the medical curriculum. It might be beneficial to organize more large-scale courses and interdisciplinary sessions, maybe also in collaboration with other departments in the medical school and beyond (eg, medical informatics) to increase the range and achieve higher participation numbers. In doing so, the relevance of digital health for students’ future daily work as physicians could also be highlighted. It is also worth discussing to what extent students’ needs regarding organization, amount of work, and implementation could be combined (eg, preferences regarding block seminars in the semester break, face-to-face sessions, or blended learning formats).

The provision of digital health education represents an important step in supporting future practicing physicians who need to be able to use and prescribe digital health interventions. By integrating the elective into medical curricula, the willingness of physicians and psychotherapists to prescribe DiGAs could be increased. In a previous study, 63% of surveyed general practitioners indicated a relatively low willingness to prescribe DiGAs [47]. This could be attributed to concerns about safety, reliability, additional workload [48], personal uncertainty [21], lack of knowledge, lack of reliable information sources [49], and a lack of evidence of effectiveness [19]. The inclusion of digital health education in medical studies could address these concerns and positively influence the already established infrastructure of DiGAs through well-designed and trusted information based on academic training.

### Limitations

The limitations of this case study are the small sample sizes and the conduction at only 1 medical school in Germany, which may restrict the generalization of the findings of the evaluation to a broader or more diverse population of medical students (eg, students with different preferences and experiences and students in more advanced semesters). We did also open the elective to further study subjects beyond medicine to broaden the perspectives and capture the interdisciplinary nature of digital health. The integration of a certain proportion of other subjects was also beneficial because there are approximately 150-200 electives per semester at our medical school that the students can freely choose from and we could only conduct the elective with at least 5 students. Nonetheless, we adhered to the requirement that the vast majority of the web-based seminars had to consist of medical students (usually at least two-thirds of the participants).

Furthermore, we did not statistically compare the evaluation outcomes (mean scores) across the semesters as the sample sizes were small and unequal. Instead, initial individual insights can serve as starting points for a broader implementation of digital health in the German curricula for medical students. In addition, there could be a potential bias due to participant self-selection—students chose the elective course themselves. Therefore, it might be possible that they already had a greater interest in the topic or were more familiar with digital health in general, which might have led to more positive evaluations.

Furthermore, the prescription of DiGAs, which was a key topic of the elective, is still unique to the German health care system, making several of the contents of the lectures and ILIAS modules for self-guided learning hardly generalizable to medical education in other countries. However, other aspects are generally important for education on digital health and app development (eg, gamification). In addition, the design thinking approach to develop app concepts is universally adaptable to electives that aim to foster digital competencies in medical education outside the context of Germany.

Another limitation is the fact that the input of lecturers and experts might have had a strong influence on the students’ focus on the development of app concepts. This could have led to a potential bias on the part of the students in terms of topics and priorities in the implementation process. However, it was essential to provide sufficient knowledge beforehand, and this enabled students to put special emphasis on factors that were relevant for them (such as gamification, adherence promotion, or data security). Furthermore, the evaluation was based primarily on students’ feedback. Thus, the possible gain of knowledge and usefulness for the students cannot really be assessed. In future electives, it could be helpful to assess pre- and postknowledge on digital health topics addressed in the elective in a standardized manner. In this project, we only gathered this information via verbal feedback in the first and last session as well as via 1 item in the anonymized survey (perceived knowledge gains). However, students’ presentations and feedback indicated that they seemed to have learned relevant aspects concerning digital health. Finally, there was no feedback from students who started the elective but chose to terminate participation or from students who did not choose the elective in the first place. These insights could have been useful to further improve the course and find out more about the generally rather low participation rate (eg, whether this was related to the course content or to organizational and time aspects).

### Implications

#### For Medical Schools

Currently, there is a discrepancy between the demand for and the benefits of DMHIs and their integration into medical education. Although medical students acknowledge the importance of digital health in enhancing core skills [50], participation in our elective was rather low. Existing research literature highlights that students and health care providers have a discernible knowledge gap in the domain of digital health. To address this, providing clear information about the benefits of such electives is crucial. A general lack of awareness, compounded by insufficient information from academic faculties, may hinder progress. In Germany, digital health courses are limited, mainly offered as electives [2]. Even though more and more medical schools participate in surveys in which they are asked whether they offer digital health courses, indicating an interest in the topic, the number of courses they actually provide appears to stagnate [51]. This discrepancy highlights the importance of defining standards and providing guidance for digital health education across medical schools. Integrating digital health into curricula has the potential to enhance future physicians’ capabilities, but specialized training is vital for navigating the digital age. Introducing digital health competencies equips aspiring clinicians with essential skills. These include fostering positive patient-physician relationships and explaining the risks and benefits. Foundational knowledge can be integrated into standard medical curricula, with electives focusing on specific in-depth knowledge and specializations [30].

#### For Teaching Staff

To successfully implement and execute courses that focus on digital health and app development, it is useful to provide enough time for the students to take in relevant aspects and knowledge (eg, regular weekly courses instead of block seminars). Regular exchange among students and between students and teaching staff also seems to be important. The use of interactive web-based learning platforms and chat programs can also improve the course (eg, ILIAS and Rocket.Chat). Involving different occupational groups and experts (eg, start-up developers and economists) facilitates networking and allows for the exposure to different perspectives and in-depth knowledge for the students. The concept itself is scalable at a larger level even though it needs staff deployment, and the focus or topics of the elective are interchangeable. Finally, it could be beneficial to collaborate with other institutes or departments to increase the range of courses related to digital health.

#### For Researchers

Further quantitative and qualitative research methods could be useful to gain more insights into the preferences and needs of medical students with regard to digital health. It might also be beneficial to conduct research concerning outcomes of learning success that goes beyond individual feedback in the form of questionnaires and open questions. Research among the general population of medical students in Germany is needed to deepen existing knowledge, further adapt elective courses, and prepare the integration of digital health into the medical curricula.

### Conclusions and Outlook

A current challenge in health care and prevention is a lack of knowledge and competencies in the field of digital health among health care providers. To increase the prescription readiness and establish digital health in the everyday lives of patients and physicians, it needs to be implemented in medical education. Further research is needed to define specific learning objectives for digital health competencies and develop detailed recommendations for their integration into medical curricula.

## Appendix

Multimedia Appendix 1 Additional prototype visualizations.

Multimedia Appendix 2 Additional results.

## Abbreviations

AI: artificial intelligence
ILIAS: Integriertes Lern-, Informations- und Arbeitskooperations-System (German for “Integrated Learning, Information and Work Cooperation System”)
DiGAs: Digitale Gesundheitsanwendungen (German for “digital health applications”)
DMHI: digital mental health intervention
HHU: Heinrich Heine University Düsseldorf
