# Supplementary material for: Enhancing Digital Health Awareness and mHealth Competencies in Medical Education: Proof-of-Concept Study and Summative Process Evaluation of a Quality Improvement Project
Source: JMIR Med Educ. 2024 Sep 20;10:e59454. doi: 10.2196/59454 (PMC11452754; doi:10.2196/59454)
Supplement: Multimedia Appendix 1 [file mededu_v10i1e59454_app1.pdf]

Multimedia Appendix 1: Additional Prototype-Visualization

Case examples: app development and concepts

Additional prototypes and mock-ups of four mobile health apps designed by students in an elective at the Medical Faculty of the Heinrich Heine University Düsseldorf, Germany*:

1. *Bruhno* (Figures S1 and S2)
2. *MeTime* (Figures S3 and S4)
3. *Dinotherapy* (Figure S5)
4. *Companion* (Figures S6 and S7)

* Further details can be found in the article.

1. *“Bruhno” app – Brustkrebs Helfer für Nebenwirkungen und Organisation (Breast cancer helper for side effects and organization)*

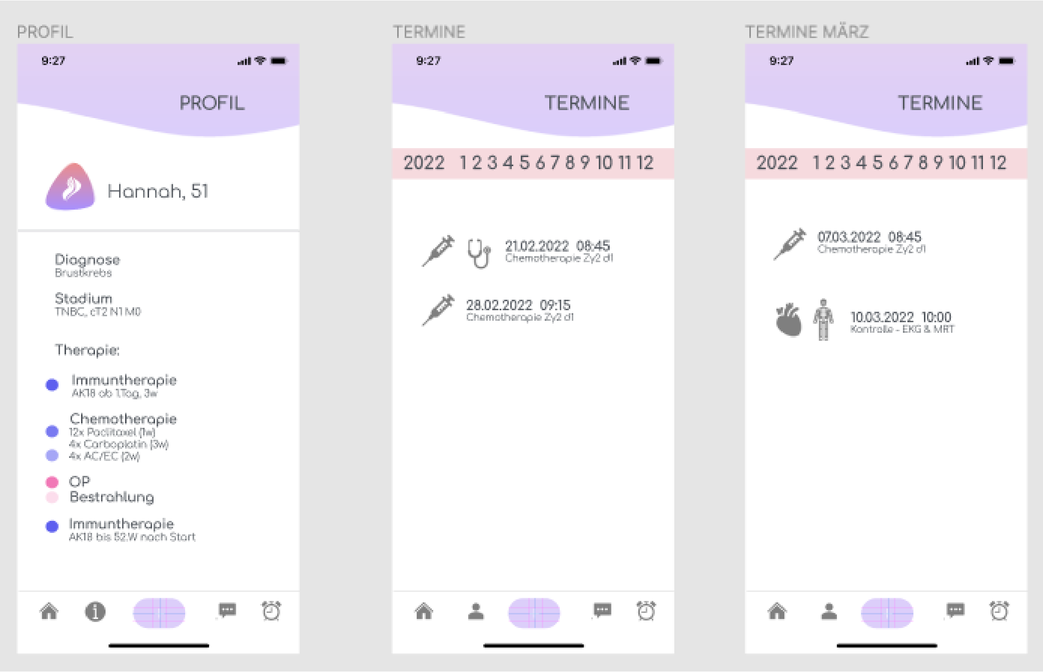

Figure S1. Mock-up of the app *Bruhno*: Personal profile (with diagnosis, stage of the disease and therapies) and upcoming appointments.

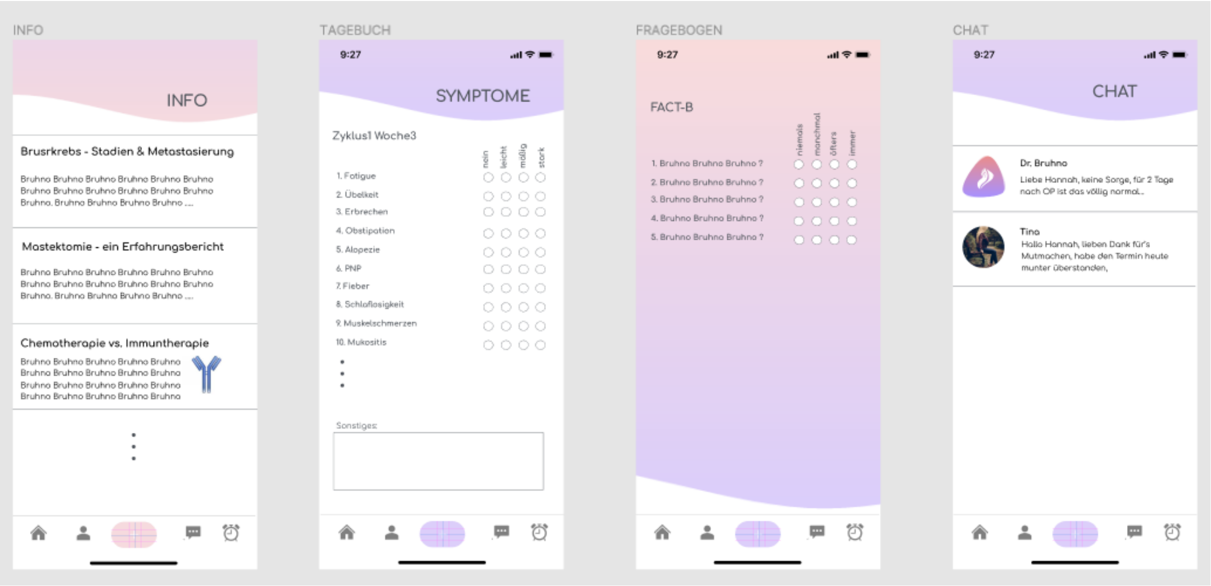

Figure S2. Mock-up of the app “*Bruhno*”: Personal information (e.g., progress report), diary, questionnaire, and chat function. Created using Figma GmbH (student license; Berlin, Germany).

2. *"MeTime" app*

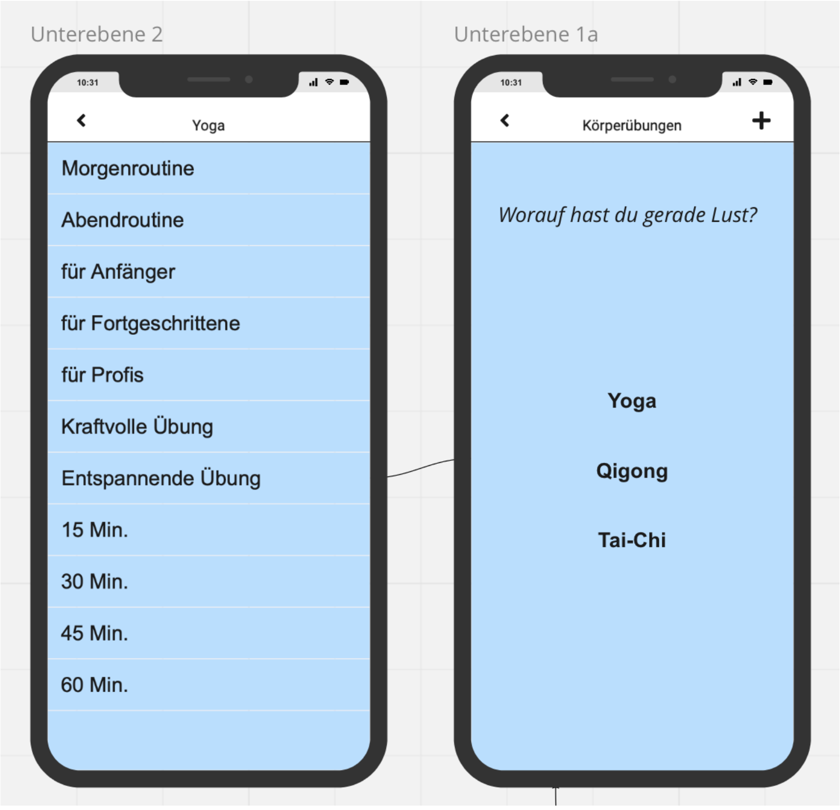

Figure S3. Mock-up of the app *MeTime*: exemplary physical and yoga exercises.

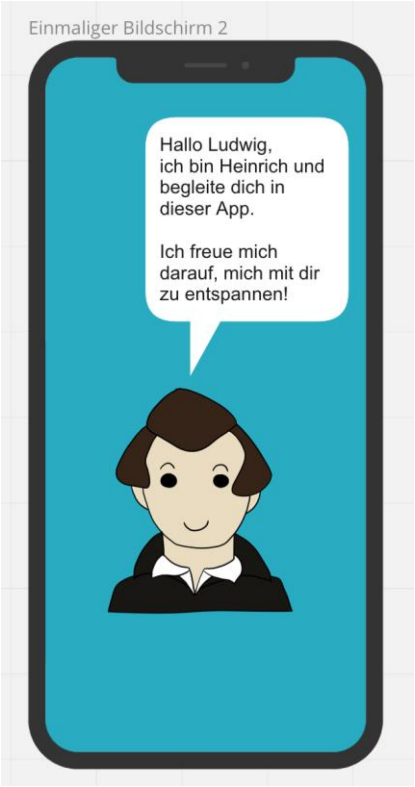

Figure S4. Mock-up of the app *MeTime*: visualization of introduction of the companion "Heinrich". Screen text (translated from German): "Hello Ludwig, I am Heinrich, and I will be accompanying you in this app. I'm looking forward to relaxing with you!".

3. *“Dinotherapy” app*

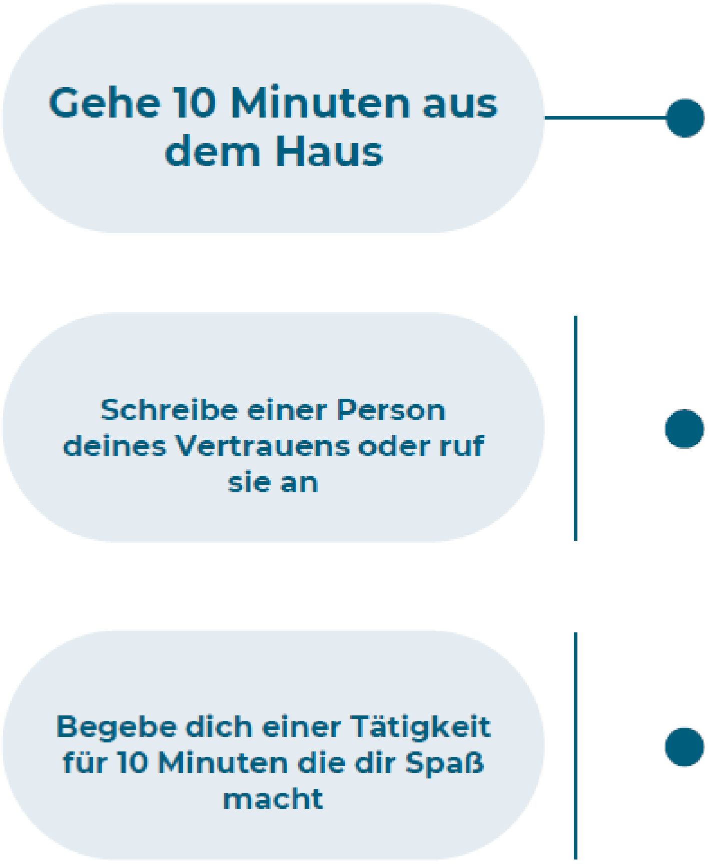

Figure S5. Mock-up of the app *Dinotherapy*: example for a daily task to improve the user’s motivation. Screen text: “leave the house for 10 minutes, write or call a person you trust, do an activity you enjoy for 10 minutes”.

4. *“Companion” app*

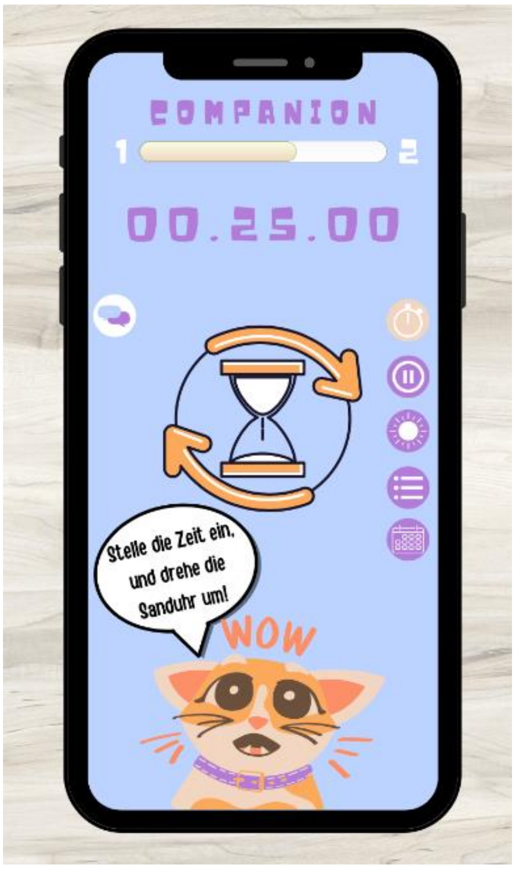

Figure S6. Mock-up of the app *Companion*: visualization of the timer for concentrated work phases.

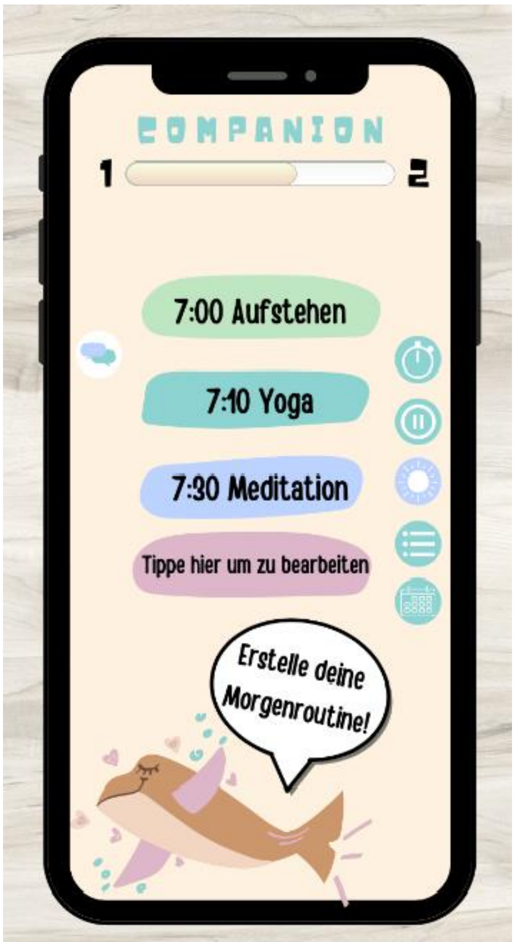

Figure S7. Mock-up of the app *Companion*: visualization of a customizable morning routine.
