# Supplementary material for: Enhancing Digital Health Awareness and mHealth Competencies in Medical Education: Proof-of-Concept Study and Summative Process Evaluation of a Quality Improvement Project
Source: JMIR Med Educ. 2024 Sep 20;10:e59454. doi: 10.2196/59454 (PMC11452754; doi:10.2196/59454)
Supplement: Multimedia Appendix 2 [file mededu_v10i1e59454_app2.pdf]

Multimedia Appendix 2: Additional Results

Quantitative Research Results

Figures S8 and S9 display the aggregated results on the ratings regarding the satisfaction with the learning module (ie, web-based seminar on the creation of app concepts by students) as well as the subjective evaluation of the quality of the digitalization of the seminar in 6 different semesters at a medical school in Germany (SS 2021-WS 2023/24).

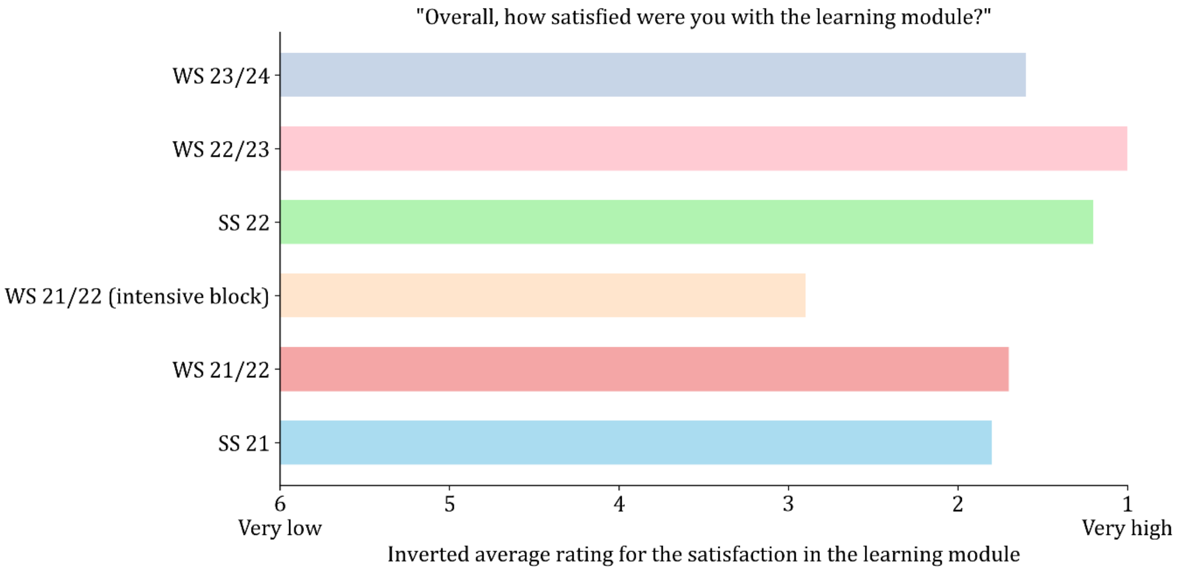

Figure S8. Assessment of students´ satisfaction with the elective subject conducted at the end of each semester. Abbreviations: SS: summer semester, WS: winter semester.

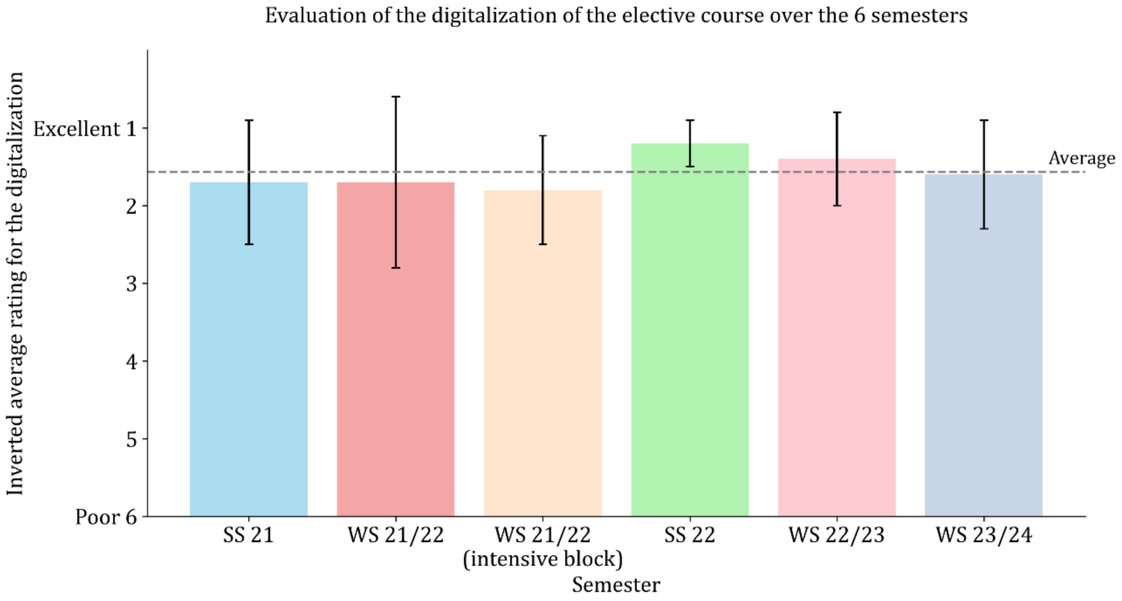

Figure S9. Evaluation of the digitalization in the elective subject over 6 courses within 5 semesters. The ratings are based on student ratings in orientation to the German grading system in education (1=excellent; 6=poor), with lower values indicating better ratings. The inversion is intended to improve readability. The bars represent the inverted mean scores, and the error bars indicate standard deviations, with higher bars now corresponding to higher digitization ratings. A dashed line indicates the average rating across all semesters. Abbreviations: SS: summer semester, WS: winter semester.
